# What Is Targeted When We Train Working Memory? Evidence From a Meta-Analysis of the Neural Correlates of Working Memory Training Using Activation Likelihood Estimation

**DOI:** 10.3389/fpsyg.2022.868001

**Published:** 2022-03-30

**Authors:** Oshin Vartanian, Vladyslava Replete, Sidney Ann Saint, Quan Lam, Sarah Forbes, Monique E. Beaudoin, Tad T. Brunyé, David J. Bryant, Kathryn A. Feltman, Kristin J. Heaton, Richard A. McKinley, Jan B. F. Van Erp, Annika Vergin, Annalise Whittaker

**Affiliations:** ^1^Defence Research and Development Canada, Toronto, ON, Canada; ^2^Department of Psychology, University of Toronto, Toronto, ON, Canada; ^3^Faculty of Medicine, Queen’s University, Kingston, ON, Canada; ^4^Department of Psychology, University of Waterloo, Waterloo, ON, Canada; ^5^Department of Psychiatry, University of Manitoba, Winnipeg, MB, Canada; ^6^Applied Research Laboratory for Intelligence and Security, University of Maryland, College Park, MD, United States; ^7^U.S. Army DEVCOM Soldier Center, Natick, MA, United States; ^8^U.S. Army Aeromedical Research Laboratory, Fort Rucker, AL, United States; ^9^U.S. Army Research Institute of Environmental Medicine, Natick, MA, United States; ^10^U.S. Air Force Research Laboratory, Wright-Patterson Air Force Base, Dayton, OH, United States; ^11^Netherlands Organization for Applied Scientific Research (TNO), Soesterberg, Netherlands; ^12^Department of Human Media Interaction, University of Twente, Enschede, Netherlands; ^13^Bundeswehr Office for Defence Planning, Federal Ministry of Defence, Berlin, Germany; ^14^Defence Science and Technology Laboratory, UK Ministry of Defence, Salisbury, United Kingdom

**Keywords:** working memory span, training, cognitive resource, meta-analysis, executive functions

## Abstract

Working memory (WM) is the system responsible for maintaining and manipulating information, in the face of ongoing distraction. In turn, WM span is perceived to be an individual-differences construct reflecting the limited capacity of this system. Recently, however, there has been some evidence to suggest that WM capacity can increase through training, raising the possibility that training can functionally alter the neural structures supporting WM. To address the hypothesis that the neural substrates underlying WM are targeted by training, we conducted a meta-analysis of functional magnetic resonance imaging (fMRI) studies of WM training using Activation Likelihood Estimation (ALE). Our results demonstrate that WM training is associated exclusively with decreases in blood oxygenation level-dependent (BOLD) responses in clusters within the fronto-parietal system that underlie WM, including the bilateral inferior parietal lobule (BA 39/40), middle (BA 9) and superior (BA 6) frontal gyri, and medial frontal gyrus bordering on the cingulate gyrus (BA 8/32). We discuss the various psychological and physiological mechanisms that could be responsible for the observed reductions in the BOLD signal in relation to WM training, and consider their implications for the construct of WM span as a limited resource.

## Introduction

Working memory (WM) is defined as “a multicomponent system for active maintenance of information in the face of ongoing processing and/or distraction” (Conway et al., 2005, p. 770). Most classic accounts of WM have conceptualized this system to be limited in *capacity*, reflecting the underlying notion that it represents a limited resource (e.g., Miller, 1956; Cowan, 2001; for a review, see Baddeley, 2003). Broadly speaking, a processing resource can be defined as “something that exists in limited supply and is responsible for the enhancing or enabling of certain cognitive processes” (Salthouse, 1990, p. 102). Within the construct of WM, *capacity* reflects individual differences in the limit of this system, indicating that people can process only a certain amount of content at any given time. Examining *why* WM capacity is limited remains an active area of research, with candidate processes (to be described further, below) including temporal decay, limitations in cognitive resources and mutual interference of WM representations, among others (see Oberauer et al., 2016).

In contrast to accounts which consider WM to be a resource-limited system which is only able to store and process a small, fixed number of items, some contemporary views have emphasized the flexibility with which information can be maintained and manipulated in WM. For example, Ma et al. (2014) reviewed a large body of behavioral and neuroimaging data to argue for alternative resource models that do not invoke a fixed limit on how many items can be stored in short-term memory (e.g., magical number 4, or magical number 7—plus or minus 2, etc.), but instead emphasize that WM capacity depends on the quality or precision with which items are processed. Such flexible resource models of WM assume that the internal representations of sensory stimuli are inherently noisy, and that this noise increases as the number of to-be-remembered items increases in memory (see Palmer, 1990; Wilken and Ma, 2004; Bays and Husain, 2008). In turn, the extent to which any given item is recalled with precision depends on the quantity of resources devoted to processing it: As this quantity increases, there is a corresponding decrease in the noise associated with the item in memory, and increased likelihood of precise recall. Consistent with such accounts, it has been shown that there is less precision in the recall of items from memory as the number of to-be-remembered items increases, and increased precision in recall as their salience or goal-relevance increases (Gorgoraptis et al., 2011). The upshot of this contemporary work is that even when resources are limited, there can be flexibility in their allocation as a function of context and goals, which can in turn impact quality as well as quantity of recall.

### Behavioral Effects of Working Memory Training

Consistent with such flexible notions of information processing in WM, there has been great interest recently in improving WM capacity, skills, and performance *via* targeted training (see Klingberg, 2012). Indeed, several largescale meta-analyses and reviews of the behavioral literature have shown that WM training can lead to *near transfer*—defined as performance improvements on short-term and WM tasks that are similar to the trained task (Morrison and Chein, 2011; Melby-Lervåg and Hulme, 2013; Redick et al., 2015; Melby-Lervåg et al., 2016; see also Soveri et al., 2017). Evidence for near transfer suggests that WM training likely targets cognitive processes that are commonly shared by most short-term memory and WM tasks, such as maintenance and updating of information. In contrast, there is little or no reliable evidence to suggest that WM training can lead to *far transfer*—defined as observing performance benefits in outcome measures that are dissimilar to the trained task in terms of structure or surface features (Perkins and Salomon, 1994; but see Au et al., 2015). There could be many reasons why reliable evidence for far transfer has not been observed. One reason could be that the untrained tasks likely recruit other capabilities in addition to WM that must also be targeted by training for benefits to be observed in performance, including perhaps other executive functions (e.g., switching and inhibition). Another possibility might be that the gains observed in WM span are due to the development of strategies that are applicable to only certain tasks but not others, or at least not to the same extent (e.g., chunking). Finally, it could also be that WM training only leads to gains in some aspects of WM span but not others (see Shipstead et al., 2014), therefore limiting its broad utility. More generally, it is likely necessary to specify the dimensions along which far transfer can occur to optimize the goodness-of-fit between what is trained and the target tasks that it is meant to transfer to (see Barnett and Ceci, 2002).

Consistent with evidence that WM training can lead to near transfer, there are also findings to suggest that WM training can lead to gains in WM capacity. For example, Harrison et al. (2013) asked participants to complete a battery of near-, moderate-, and far-transfer tasks at baseline, followed by 20 sessions of training that consisted of one of following three conditions: Participants in the complex-span training condition completed adaptive versions of the operation-span and symmetry-span tasks during each session, whereas participants in the simple-span training condition completed two adaptive simple span tasks. In turn, the control condition consisted of participants who trained on an adaptive visual search task only. The same battery of near-, moderate-, and far-transfer tasks were completed after training. In terms of near transfer, the complex- span training group exhibited improvements on the rotation- and reading-span tasks, even though both contained different distractor tasks and different to-be-remembered items than the training tasks. Both the complex-span and simple-span training groups also showed improvement on the running-letter-span and running-spatial-span tasks. Because the same to-be-remembered stimuli were used for the training and running-span tasks, this improvement could be attributable to either an increase in WM capacity or learning of stimulus-specific strategies for remembering letters and matrix locations. In terms of moderate transfer, both the complex-span and simple-span training groups showed improvement on the secondary memory component of immediate free recall. In terms of far transfer, no group exhibited any gain in fluid intelligence. These results suggest that WM training can lead to improvement in WM span, although it is important to remember that one can observe such improvements without necessarily improving WM capacity at the construct level. This is because not all of the variance in WM span task performance reflects WM capacity, but can instead reflect other factors related to the performance (e.g., strategies, ability to chunk letters, and random error) (Kane et al., 2004; see Harrison et al., 2013) and beyond (e.g., stress, fatigue, and sleep loss). As such, when improvement in WM capacity is observed, care must be exercised in interpreting what has been targeted and improved by training (see also Vartanian et al., 2016, 2021).

### Process Specificity and the Brain

Although researchers have begun to gain traction on some of the processes and mechanisms underlying behavioral performance improvements associated with WM training—including its possible moderators (see Jaeggi et al., 2014; Au et al., 2015)—relatively less is known about its neural correlates (see Buschkuehl et al., 2012). Nevertheless, a number of insights have begun to emerge based on the available literature. First, there is good reason to believe that whether transfer does or does not occur depends in part on *process specificity*—defined as the extent to which the specific cognitive process affected by the training task also underlies performance on the untrained task (Eriksson et al., 2016). Examples of such processes include the storage of information, suppression of distractors, and updating of information (see Flegal et al., 2019). Process specificity is important at the neural level because the greater the functional similarities between the trained and untrained tasks, the greater the likelihood that the sets of brain regions underlying those tasks will also overlap. In this sense, brain imaging studies are useful because they can reveal possible neural mechanisms whereby training-related improvements and transfer could occur (Klingberg, 2010; Buschkuehl et al., 2012).

For example, Dahlin et al. (2008) examined participants’ brain activity using functional magnetic resonance imaging (fMRI) before and after a 5-week regimen of WM training. Neural data were obtained to assess training-related changes in brain activity. Training consisted of a letter memory task that focused specifically on updating of information in WM. The experimenters administered three tasks while participants underwent fMRI: The letter memory task, the *n*-back, and the Stroop task—the latter two being the transfer tasks. Importantly, both the letter memory task and the *n*-back task involved updating of information in WM, whereas the Stroop task did not. Not surprisingly, all three tasks engaged the well-established fronto-parietal WM system. In terms of the two transfer tasks, the investigators reasoned that if transfer hinges on a shared fronto-parietal network, then it should be observed for both the *n*-back task and the Stroop task—because both share activation in that region with the letter memory task. However, if transfer hinges specifically on updating of information in WM and is associated with shared activity in the striatal updating network, then it should be observed for the *n*-back task only. Indeed, the results supported the latter prediction, demonstrating that transfer occurs if the training task targets the same cognitive process and/or mechanism that underlies the transfer task—in this case updating of information in WM.

### Increases and Decreases in Brain Activation

A second finding that has emerged from neuroimaging studies is that WM training can be correlated with both increases as well as decreases in brain activation, although the reasons behind this variability in the observed results are not well-understood. For example, in his early review of this literature Klingberg (2010) noted a pattern such that studies that involved short periods of WM training (<3 h) had been shown to result in decreased brain activity, whereas long periods of WM training had been shown to result in an admixture of both increased and decreased brain activity. Klingberg (2010) proposed that the decreases in activation could have occurred because of a number of different processes taking place, including strategy learning, priming during encoding, and time-on-task effects—all of which have been shown to be correlated with reductions in brain activation (see also Brouwer et al., 2014). In turn, during longer training regimens these reductions would be co-occurring with increases in WM capacity, which would in turn be correlated with activity in the intraparietal cortex, middle and superior frontal gyri, and the caudate nucleus. However, in their own review of largely the same literature on the neural effects of WM training, Buschkuehl et al. (2012) called for additional data to understand the impact of WM training on neural function. Specifically, they reviewed evidence from several studies to demonstrate that WM training was associated with decreases in brain activation in many fMRI studies, suggesting that perhaps brain function can become more efficient with increased practice and expertise. Given that brains are metabolically expensive, the ability to perform tasks to the same or improved level with less energy expenditure would represent a significant adaptive benefit.

Dahlin et al.’s (2009) review of the neuroimaging studies of WM training reached a conclusion quite similar to Buschkuehl et al. (2012) in attempting to interpret patterns of neural activation and deactivation. Namely, they noted that the central executive component of Baddeley’s (1996) model of WM has been linked strongly to the fronto-parietal system.^1^ Although greater activation in this system as a function of WM training can be attributed to either the recruitment of additional cortical units with practice or the strengthening of the blood oxygen level-dependent (BOLD) response within a specific region, a far more common observation is a reduction in activation in this system in association with WM training. Such reductions could mean that the task was initially difficult and required resources from the central executive, but with practice became less difficult or required less conscious thought and thus required fewer resources—and by extension less fronto-parietal involvement.

Interestingly, the opposite pattern was perceived in subcortical areas such as the basal ganglia where brain activation was far more likely to increase following WM training. Dahlin et al. (2009) argued that such increases in activation in subcortical areas could in turn reflect the strengthening of the specific skills in association with training (e.g., updating of information in WM). This two-pronged view suggests that during the early phases of learning the prefrontal cortex likely exercises cognitive control for the purpose of new rule and skill acquisition, whereas over time, when the previously novel rules and skills have been learned, the frontal lobes become less engaged and the acquired rules and skills are implemented by other neural systems (see Packard and Knowlton, 2002; Poldrack et al., 2005). Thus, Dahlin et al. (2009) interpreted “the decreased cortical activation as an indication of more automatized task performance following training, and the increased striatal activation as a change in the underlying skill” (p. 411). This interpretation is also consistent with the idea that rather than being a unitary construct, training can encompass the acquisition of new mental operations or shortcuts as well as reducing inefficiencies in existing processes. For example, Bryant and Niall (2020) characterized three approaches to cognitive optimization that are analogous—increasing the power of a cognitive capability, increasing the effect one can derive from an existing level of capacity, and providing external devices to perform cognitive tasks to reduce the need for using cognitive capabilities. Training might be viewed in a similar fashion—increasing the capacity of WM, making WM more efficient, or off-loading some functions of WM to other cognitive capacities. In turn, these effects can be associated with variations in the structures and directions of BOLD activity change in relation to WM training.

A fundamental problem when assessing this body of work concerns how to interpret the changes in the BOLD signal observed in relation to WM training. For example, in several cases to date, reductions in the BOLD signal due to training have been interpreted as reflecting increased efficiency of neural function. Poldrack (2015) has argued convincingly that such an interpretation is unjustified because a reduction in the BOLD signal does not necessarily mean that there is less energy expenditure for conducting the same task. Indeed, a reduction in the BOLD signal can be observed because a different set of cognitive processes and/or neural computations are being performed—neither of which means that there is reduced energy expenditure for the same amount of work. As noted by Poldrack (2015), one could argue for neural efficiency if the same neural computation were being performed with identical time and intensity, but with different metabolic expenditure due to factors such as amount of transmitter release, nature of neurovascular coupling, or the degree to which the neural computations draw on oxidative vs. non-oxidative metabolism. However, such inferences require information about metabolism at the cellular level, which the BOLD signal does not provide (Logothetis, 2008). Constantinidis and Klingberg (2016) came to a similar conclusion when interpreting the literature on the neuroscience of WM training, as the changes in brain activation could be due to many physiological factors including the number and/or the firing rate of the neurons during maintenance of representations in WM, among others. This prompted them to note that “A cautious interpretation is thus that these fMRI studies point to the areas of change but do not inform us about the underlying cellular mechanisms” (p. 444). Nevertheless, localizing where the changes occur and the direction in which they occur is a necessary first step for understanding the structures whose function is impacted by training, although subsequent research will be necessary to understand precisely why the changes have occurred, and the extent to which they reflect variations in metabolic expenditure at the cellular level.

### Aims of Present Meta-Analysis

Our meta-analysis had three aims. The first aim was to reveal brain structures that are activated reliably across studies as a function of WM training. To this end, we employed the Activation Likelihood Estimation (ALE) approach, which is a widely adopted coordinate-based platform for the quantitative meta-analysis of neuroimaging data (Eickhoff et al., 2012). To address this aim, we specifically restricted our focus to studies that involved pre-test and post-test assessments of WM performance with fMRI, and training regimens involving a WM task. We are aware of three earlier meta-analyses of the literature on the neural bases of WM training, with different scopes and aims than ours. First, Li et al. (2015) investigated the neural correlates of WM training in healthy adults and patients with schizophrenia. Next, Salmi et al. (2018) investigated the neural correlates of WM training in healthy adults, but also included studies in which the target fMRI task was not necessarily a WM task (e.g., multitasking, divergent thinking, etc.). In turn, Pappa et al. (2020) focused exclusively on studies that utilized a WM updating task as the training task (rather than a maintenance task, etc.) to achieve greater homogeneity across studies in terms of the specific process that was being trained. All three meta-analyses included data from elderly samples. Although these meta-analyses have made valuable and important contributions to our understanding of the neural bases of WM training, we believe that the present meta-analysis fills a unique niche in the literature. First, we focused exclusively on samples of neurologically healthy adults having a mean age of <65 years, given the well-established finding that older adults display overactivation in functional brain imaging studies, likely as a compensatory mechanism against age-related decline in cognition (for review, see Reuter-Lorenz and Cappell, 2008; see also Cabeza et al., 2018; Tagliabue and Mazza, 2021). We reasoned that focusing on young-to-middle aged adults would reduce some of the heterogeneity in the findings due to the age-related differences in brain activation. Second, we focused exclusively on studies that has used a WM task both for training and for pre- and post-testing. The reason for this decision was to reduce heterogeneity in the tasks under consideration by focusing only on tasks that target WM function. We reasoned that by virtue of focusing on neurologically healthy non-senior adults who were trained and tested (pre- and post-training) exclusively on WM tasks, we would be in a position to examine whether training on any WM task can reliably impact brain function in regions of the brain that underlie WM in target tasks. Of particular interest were regions in the fronto-parietal network that have been consistently linked to performance and individual differences in this capacity (Wager and Smith, 2003; Owen et al., 2005; Darki and Klingberg, 2015), as well as subcortical systems such as the basal ganglia (Eriksson et al., 2016).

The second aim of our meta-analysis was to examine whether there are differences in brain regions that exhibit activation increases vs. decreases as a function of WM training (i.e., the directionality of training effects in the BOLD response). Indeed, one of the distinguishing features of individual studies to date has been the heterogeneity in the direction of change noted in brain activation following WM training, with some studies reporting exclusively increases or decreases in activations, whereas others have reported changes in both directions in different structures. As noted by Dahlin et al. (2009) in their review of this literature, the results “support the views that training does not result in a monotonic increase or decrease in neural activity…, and that training-related activation changes are not restricted to an isolated part of the brain. To better understand the neural reorganization that takes place after training, it is critical to identify neural networks underlying these activity changes” (p. 410). To address this second aim, we distinguished between foci that have shown increases vs. decreases in activation, aiming to highlight the reliability of the directionality of the differences in response to WM training. Notably, all three meta-analyses of WM training to date have revealed an admixture of activity increases and decreases in the brain (Li et al., 2015; Salmi et al., 2018; Pappa et al., 2020). We were keen to examine whether a similar pattern would arise when the scope was limited to neurologically healthy non-elderly adults who were trained and tested (pre- and post-training) exclusively on WM tasks.

The third aim of our meta-analysis focused not on the neural data, but instead on behavioral data collected in a subset of the fMRI studies under examination that had administered WM span tasks pre- and post-training. This is because from a theoretical perspective, we were particularly interested in the impact of WM training on WM span. Therefore, aside from conducting the meta-analysis of fMRI data to address the first two aims of the study, we also conducted a descriptive review of the subset of studies that had administered WM span tasks pre- and post-training to examine the reliability of transfer from WM training to WM span, and to examine whether there are specific features of training and testing that increase the likelihood of that transfer. This descriptive review was meant to supplement the core meta-analysis of the fMRI data by shedding light on factors that facilitate transfer from WM training to WM span, and what the implications might be for models of WM that treat WM span as a limited resource.

## Method

### Literature Search

**FIGURE 1:**
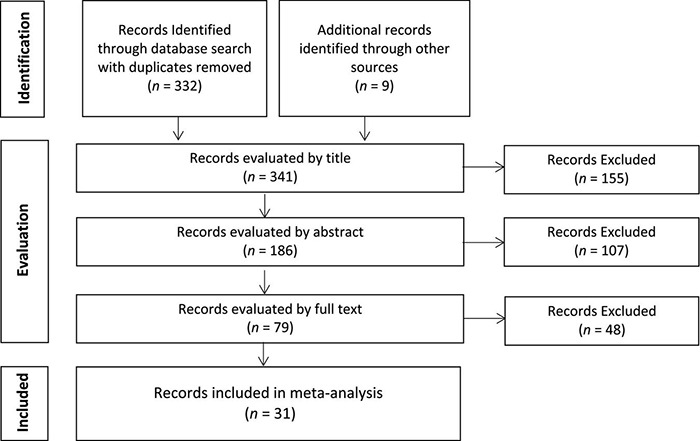
Flow diagram for literature search.

The identification of articles relating to WM training was conducted by a series of Boolean searches of PsychINFO, PubMed, and Web of Science databases last updated in January 2022. The following keywords were used: “working memory training,” “brain training,” “cognitive training,” “fMRI,” and “PET.” Furthermore, we examined review papers, past meta-analyses, and reference sections for additional studies. Our search yielded 341 references. These references were subsequently screened based on (a) article and journal title information, (b) abstract information, and (c) full-text evaluation (see Figure 1). Ultimately, this yielded 32 studies (reported in 31 unique publications) for the meta-analysis.

### Selection Criteria

The articles were screened for neurologically healthy participants. In cases where a neurologically healthy control group was included as a comparison condition for a patient group, the data from the former group were included in the meta-analysis if separate results had been reported (*n* = 2), or by contacting the authors to obtain results only from the neurologically healthy control sub-group (*n* = 1). We focused exclusively on studies that reported data from samples with a mean age of <65 years. All articles included a WM training regimen, although the specific training task varied across studies. Furthermore, in each case the pre- and post-test measures were also WM tasks. In some cases, the pre- and post-test WM measures were identical to the WM training task, whereas in others it was a different WM task that was implemented for training vs. pre- and post-testing (Table 1).

**TABLE 1:** List of 32 studies included in the meta-analysis.

Reference	Raw coordinates	Training task	Target task	Frequency (sessions)	Duration (min)
Aguirre et al., 2019	MNI	Adaptive n-back task	n-back task	4	60
Ando et al., 2007	Tal	Visuospatial WM	Visuospatial WM	105	?
Ando et al., 2009	Tal	Visuospatial WM	Visuospatial WM	210	?
Buschkuehl et al., 2014	MNI	Visuospatial n-back task	Visuospatial n-back task	7	20
Chang et al., 2017	Tal	Cogmed	2 back	20–25	30–40
Clark et al., 2017a	MNI	Lumosity visuospatial n-back task	Lumosity visuospatial n-back task	30	20
Dahlin et al., 2008 (Exp. 1)	MNI	Multimodal WM training	Letter memory, 3 back	15	45
Emch et al., 2019	MNI	Adaptive n-back task	n-back	32	?
Flegal et al., 2019	MNI	Adaptive visuospatial and visuo-verbal WM tasks	Visuospatial WM	10	50
Gaab et al., 2006	Tal	Pitch memory	Pitch memory	5	60
Garavan et al., 2000	Tal	Visuospatial WM	Visuospatial WM	1	32*
Jansma et al., 2001	Tal	Sternberg	Sternberg	1	45
Jolles et al., 2010	MNI	Verbal WM	Verbal WM	10.5	25
Kirschen et al., 2005	Tal	Verbal WM	Verbal WM	1	12
Koch et al., 2006	Tal	Sternberg	Sternberg	1	24*
Koch et al., 2007	Tal	Sternberg	Sternberg	1	24*
Landau et al., 2004	MNI	Face recognition task	Face recognition task	1	30
Miró-Padilla et al., 2018	MNI	Adaptive n-back task	n-back task	4	50
Miró-Padilla et al., 2020	MNI	Adaptive n-back task	Auditory, arithmetic WM	4	50
Moore et al., 2006	MNI	Simultaneous match to sample, delayed recognition, family placement, family discrimination	Match to sample	7	90
Olesen et al., 2004 (Exp. 1)	Tal	Visuospatial WM, backwards digit span, letter span	Visuospatial matching task	20–30	35–45
Olesen et al., 2004 (Exp. 2)	Tal	Visuospatial WM tasks: grid, grid rotation, 3D grid	Visuospatial matching task	25	35–45
Opitz et al., 2014	Tal	Adaptive n-back	Orthographic task (Chinese character learning)	14	40*
Ramsey et al., 2004	MNI	Verbal matching	Verbal matching	1	21
Sayala et al., 2006	Tal	Delayed object/spatial recognition	Delayed object/spatial recognition	1	30
Schneiders et al., 2011	Tal	Adaptive n-back	Visual n-back	8–10	50
Schneiders et al., 2012	Tal	Auditory adaptive n-back	Auditory and visual WM	8	50
Schweizer et al., 2013	MNI	Affective dual n-back	Affective dual n-back	18–20	20–30
Thompson et al., 2016	MNI	Adaptive n-back or multiple object tracking	Dual n-back	20	40*
van Raalten et al., 2008	MNI	Sternberg	Sternberg	1	25
Wagner et al., 2021	MNI	Dual n-back	Word order recognition task	40	30
Zimmer et al., 2012	MNI	Change Detection task	Change Detection task	12	?

*WM, working memory; ?, not reported; Exp., experiment.*

*To the best of our calculations based on reported data.

All selected studies included neuroimaging data collected prior to and following WM training (i.e., pre- and post-test). In cases where post-test neuroimaging data were collected at two time points following the termination of WM training (e.g., immediately after training and again >1 month after training), we focused on the time point nearest to the termination of training (i.e., immediately after training). This allowed for a direct comparison of post-test data across studies using immediate vs. immediate *and* delayed methodologies, eliminating this potential confound. All the studies reported voxel-wise, whole brain data which reported foci in 3D coordinate space (i.e., not ROI analysis). In cases where the performance of an experimental group (i.e., WM training) was compared to a control group (i.e., active or passive control) at pre- and post-test time points, we selected the results of the Group × Time interaction effect for analysis. In cases where only the results of the training group were available/reported at pre- and post-test, we included in our analysis the coordinates associated with the simple main effect of training. In both cases above, if the authors reported results separately for different levels of difficulty of the same task (e.g., 3-back vs. 2-back for n-back at post-test compared to 1-back at pre-test), we selected the contrast that isolated the neural correlates of the more difficult level (i.e., 3-back at post-test compared to 1-back at pre-test rather than 2-back at post-test compared to 1-back at pre-test). In total, 31 articles that included 32 studies met the criteria and were included in the meta-analysis, including data from 813 participants and 385 foci (Table 1).

### Activation Likelihood Estimation

ALE is a quantitative meta-analysis technique that compares activation likelihoods calculated from observed activation foci with a null distribution of randomly generated activation likelihoods. It pools peak activation coordinates across studies that have investigated an effect of interest (Laird et al., 2005). These coordinates must be spatially renormalized to a single template. For this meta-analysis, all coordinates were renormalized to MNI space using the icbm2tal transformation (Lancaster et al., 2007) implemented in the GingerALE 3.0.2 toolbox (Research Imaging Center of the University of Texas Health Science Center, San Antonio, TX).^2^ The resulting coordinates were used to generate “activation likelihoods” for each voxel in the brain. For each focus, ALE computes each voxel as a function of its distance from that focus using a three-dimensional Gaussian probability density function centered at its coordinates. This generates vectors of values for each voxel representing probabilities of belonging to a specific focus. These values are assumed to be independent such that the existence of one focus does not give information about whether another focus will occur. The vector values are combined with the addition rule for log-probabilities, yielding ALE statistics. Thus, the ALE statistic represents the probability of a certain voxel to belong to any of the included foci. Significance tests are conducted by comparing the ALE statistic in each voxel with a null distribution, generated *via* repeatedly calculating ALE statistics from randomly placed activation foci. This null distribution is then used to estimate the threshold based on a given cut-off. Finally, a cluster threshold (i.e., minimum spatial extent of significant contiguous clusters) can be applied. As recommended in Eickhoff et al. (2016), we conducted our analyses based on a cluster-level family-wise error (FWE) correction, which involves using an uncorrected cluster-forming threshold (*p* < 0.001) and employing a cluster-extent threshold (*p* < 0.05) that controls the chance of observing a cluster of that size if foci were randomly distributed—implemented in GingerALE 3.0.2 (Eickhoff et al., 2017). We used 1,000 thresholding permutations.^3^

## Results

### Omnibus Analysis

**FIGURE 2:**
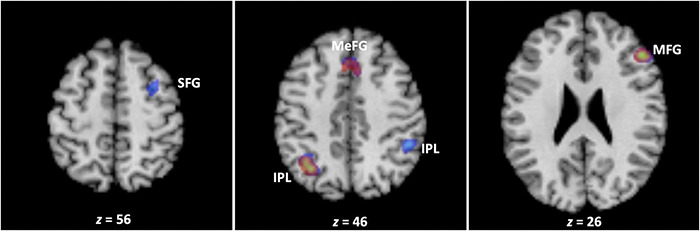
The neural correlates of working memory training. Across all studies, working memory training engaged clusters encompassing the left inferior parietal lobule (BA 40), right middle frontal gyrus (BA 9), and medial frontal gyrus bordering on the cingulate gyrus (BA 6/32) (depicted in red). In turn, working memory training was associated with decreases in brain activation in clusters encompassing the bilateral inferior parietal lobule (BA 39/40), middle (BA 9), and superior (BA 6) frontal gyrus, and medial frontal gyrus bordering on the cingulate gyrus (BA 8/32) (depicted in blue) (see text, Tables 2, 3). The transparencies of the activations are set to 50% to reveal three areas of overlap in the left inferior parietal lobule (BA 40), right middle frontal gyrus (BA 9), and medial frontal gyrus bordering on the cingulate gyrus (BA 6/32). IPL, inferior parietal lobule; MFG, middle frontal gyrus; SFG, superior frontal gyrus; MEFG, medial frontal gyrus.

The results of the omnibus analysis spanning all 32 studies revealed that WM training was associated with the involvement of the fronto-parietal system encompassing clusters in the left inferior parietal lobule (BA 40), right middle frontal gyrus (BA 9), and medial frontal gyrus bordering on the cingulate gyrus (BA 6/32) (Figure 2 and Table 2).

**TABLE 2:** The neural correlates of working memory training across all studies.

Area	BA	Center	Spatial extent of cluster	Size	Contributing studies
Inferior parietal lobule	39	−35, −59, 46	−42, −66, 40 to −28, −50, 52	1,936	Ramsey et al., 2004; Kirschen et al., 2005; Moore et al., 2006; van Raalten et al., 2008; Thompson et al., 2016; Miró-Padilla et al., 2018; Aguirre et al., 2019
Medial frontal gyrus	6/32	1, 25, 43	−8, 16, 38 to 10, 32, 48	1,880	Garavan et al., 2000; Moore et al., 2006; Sayala et al., 2006; Koch et al., 2007; Thompson et al., 2016; Miró-Padilla et al., 2018; Aguirre et al., 2019
Middle frontal gyrus	9	48, 33, 28	42, 26, 22 to 56, 38, 32	1,264	Olesen et al., 2004; Moore et al., 2006; Koch et al., 2007; Schneiders et al., 2011; Thompson et al., 2016; Aguirre et al., 2019

*Number of studies = 32, number of participants = 813, number of foci = 385. The areas have been listed in order of decreasing cluster size.*

*BA, Brodmann Area; Size, cluster size in mm^3^, coordinates are reported in MNI space.*

### Increases vs. Decreases in Activation

Next, we separated the 385 foci based on whether they had been reported as increases (176) or decreases (209) in activation in previous studies, and conducted the meta-analysis separately for each group of foci. The results demonstrated that WM training was associated with decreases in brain activation in clusters within the fronto-parietal system that underlie WM, encompassing the bilateral inferior parietal lobule (BA 39/40), middle (BA 9) and superior (BA 6) frontal gyrus, and medial frontal gyrus bordering on the cingulate gyrus (BA 8/32) (Figure 2 and Table 3). In contrast, the analysis of foci which had exhibited increases in activation in previous studies did not reveal any cluster associated with WM training.^4^

**TABLE 3:** Clusters exhibiting reduced brain activation in relation to working memory training.

Area	BA	Center	Spatial extent of cluster	Size	Contributing studies
Inferior parietal lobule	39	−34, −58, 45	−42, −66, 38 to −26, −50, 52	1,888	Ramsey et al., 2004; van Raalten et al., 2008; Zimmer et al., 2012; Thompson et al., 2016; Miró-Padilla et al., 2018; Aguirre et al., 2019
Medial frontal gyrus	8/32	1, 27, 42	−8, 16, 38 to 8, 32, 46	1,448	Sayala et al., 2006; Koch et al., 2007; Thompson et al., 2016; Miró-Padilla et al., 2018; Aguirre et al., 2019
Superior frontal gyrus	6	29, 4, 56	24, −4, 50 to 34, 12, 66	1,352	Garavan et al., 2000a; Sayala et al., 2006; Schneiders et al., 2011; Miró-Padilla et al., 2018; Aguirre et al., 2019
Middle frontal gyrus	9	49, 33, 28	42, 26, 24 to 56, 38, 34	1,328	Olesen et al., 2004; Koch et al., 2007; Schneiders et al., 2011; Thompson et al., 2016; Aguirre et al., 2019
Inferior parietal lobule	40	48, −42, 44	42, −48, 38 to 56, −38, 48	960	Koch et al., 2006; Schneiders et al., 2011, 2012; Miró-Padilla et al., 2018

*Number of studies = 25, number of participants = 648, number of foci = 209. The areas have been listed in order of decreasing cluster size.*

*BA, Brodmann Area; Size, cluster size in mm^3^, coordinates are reported in MNI space.*

*Note that no cluster exhibited increased brain activation in relation to working memory training (see text).*

### Impact of Training on Working Memory Span: Behavioral Results

Of the 32 fMRI studies included in the present meta-analysis, we identified a subset of seven studies that had administered WM span tasks before and after training. Importantly, those measures were not necessarily the tasks that were administered in the fMRI scanner before and after WM training, but were more commonly included as part of the larger set of neuropsychological measures to assess near and far transfer effects from WM training to other outcome measures. Nevertheless, a descriptive review of those studies is useful for examining the extent to which WM training can transfer to measures of WM span—both simple and complex. Measures of simple WM generally involve presenting participants with a list of to-be-remembered items (e.g., letters, digits, or words) which they must subsequently recall in the correct serial order (e.g., forward or backward) (see Unsworth and Engle, 2006). As such, span subscales from the Wechsler Adult Intelligence Scale—Revised (WAIS-R: Wechsler, 1981) can be considered measures of simple WM span. Chang et al. (2017) administered the WAIS-R Digit-Span and Spatial-Span tasks to participants in the adaptive or non-adaptive WM training groups before and after training. The results demonstrated a Group (Training vs. Control) × Time (pre- vs. post-training) interaction on both Digit Span and Spatial Span such that the (adaptive) WM training group registered significantly greater gains on both measures than did the non-adaptive control group. Olesen et al. (2004, Experiment 2) administered the WAIS-R Digit Span task to participants before and after a 5-week regimen of visuospatial WM training, observing significant post-training gains compared to baseline. Emch et al. (2019) administered the German version of the WAIS, the Hamburg-Wechsler-Intelligenztest für Erwachsene—Revision (HAWIE-R; Lutz et al., 1991) digit span sub-test (forward and backward versions) (Molz et al., 2010) to experimental and control participants before and after training. The HAWIE-R digit span sub-test requires one to repeat up to nine numbers in the same order as read aloud by the examiner (forward version), and afterward in reverse serial order (backward version). They observed a Group (Training vs. Control) × Time (pre- vs. post-training) interaction effect, such that there was a performance increase in the experimental group and a performance decrease in the control group. In contrast, Jolles et al. (2010) did not observe WM training-related gains in simple WM span as measured by the WAIS-R. Specifically, they administered the WAIS-R Digit Span task to participants who either trained on a WM task or were in a passive control condition before and after training, and did not observe a Group (Training vs. Control) × Time (pre- vs. post-training) interaction effect. Rather than administering the WAIS-R, Dahlin et al. (2008, Experiment 1) administered a different simple WM span measure referred to as “Letter Memory,” which consisted of ten lists of serially presented letters (A-D) of varying length (7, 7, 9, 9, 11, 13, 9, 15, 13, 15). The task was to recall the last four letters as quickly as possible following the termination of the presentation. The results demonstrated a Group (Training vs. Control) × Time (pre- vs. post-training) interaction such that the (updating) WM training group registered significantly greater gains in Letter Memory than did the control group.

In turn, some of the studies administered measures of complex WM span before and after WM training. As noted by Unsworth and Engle (2006), like simple span tasks, complex span tasks also require participants to recall a set of to-be-remembered items in their correct, but in addition some form of processing activity is interleaved between the to-be-remembered items. For example, Clark et al. (2017a) administered the Automated Operation Span Task (AOSPAN: Unsworth et al., 2005) and the WAIS-R Digit Span task to their participants who were randomized to either the WM training or active control condition at pre- and post-test (see Clark et al., 2017b). The AOSPAN is “a complex measure of WM which requires participants to remember the sequential ordering of presented stimuli while carrying out simple mathematic problems as a distraction” (Clark et al., 2017b, p. 8). The Group (Training vs. Control) × Time (pre- vs. post-training) interaction was not observed for either outcome measure. In turn, Flegal et al. (2019) administered complex WM span measures involving verbal stimuli with the AOSPAN (Unsworth et al., 2005) and involving visual stimuli with a change localization (Gold et al., 2006) version of the Change Detection task (Luck and Vogel, 1997). Here, too, Group (Training vs. Control) × Time (pre- vs. post-training) interactions were not observed. However, it is important to note those two WM span tasks were selected specifically because they target the executive function of updating *without* changing the demand on WM capacity itself. For that reason, the fact that training-related improvements in WM updating performance did not transfer to complex WM span measures was not surprising.

## Discussion

This meta-analysis examined the neural correlates of WM training, with three aims in mind. Below, we will discuss the results with respect to each aim in a separate subsection.

### General Neural System Sensitive to Working Memory Training

Based on a substantial body of evidence linking performance and individual differences in WM tasks to the fronto-parietal system, we had predicted that this system would be modulated by WM training across studies. This prediction was confirmed with respect to the omnibus analysis involving all studies (Table 2 and Figure 2). As noted by Salmi et al. (2018) in their meta-analysis of a largely overlapping set of studies of WM training, “current brain imaging evidence does not provide evidence of areas that would be sensitive to learning *per se* but rather emphasizes the modulation of the core systems” (p. 117). It appears that the same inference can be drawn from the present meta-analysis, focused as it was on neurologically healthy non-senior adults that were tested and trained on WM tasks exclusively. There is evidence to show that the posterior cortices are the primary site where WM representations are stored and rehearsed, and that the frontal lobes become important contributors to the process when there is interference during a retention interval (Jonides et al., 2005), or a need for top-down regulation of stored content (Lara and Wallis, 2015). The present results suggest that WM training might have a modulatory effect, both on brain regions that store information as well as those that act on stored memory representations.

Interestingly, however, subcortical structures, such as those in the basal ganglia, did not exhibit involvement in WM training, despite the fact that they have been regularly engaged by WM tasks (Eriksson et al., 2016). This could perhaps be explained by the dissociation noted by Dahlin et al. (2009) regarding the involvement of the fronto-parietal system vs. subcortical regions in WM training. Namely, they noted that whereas the fronto-parietal system may play a more central role in the executive aspects of WM training, the subcortical regions may play a more critical role in the acquisition of skills during WM. Because many different types of tasks emphasizing different types of skills were employed for WM training across studies (Table 1), the variation in the specific skills targeted by training might have engaged different subcortical regions, thereby not coalescing in a shared subcortical region across studies. Indeed, there has even been some variation in previous meta-analyses of WM training studies in terms of the engagement of subcortical structures. For example, Li et al. (2015) did not report the reliable engagement of subcortical regions in WM training, whereas subcortical regions did emerge in the meta-analyses conducted by Salmi et al. (2018) and Pappa et al. (2020). Focusing strictly on WM updating studies, Pappa et al. (2020) reported consistent fronto-parietal activity decreases, but an admixture of activity increases and decreases in subcortical regions. Reviewing specific studies in the area, they noted that subcortical regions were more likely to be engaged if the training regimen had specifically involved a WM updating task than other varieties of WM tasks. As such, they argued that subcortical systems are more likely to be engaged by WM training if the task necessitates goal-directed flexibility—a hallmark of updating tasks. In support of this view, Pappa et al. (2020) reviewed theoretical frameworks according to which subcortical systems are hypothesized to play an important role in exhibiting goal-directed flexibility in behavior, in part *via* their interplay with the prefrontal cortex (Cools and D’Esposito, 2011; Nyberg and Eriksson, 2016). In turn, Salmi et al. (2018), who explored differences in the neural systems that support WM training vs. perceptual-motor learning, noted that the striatum was involved in both processes. This suggests that rather than making a unique contribution to WM *per se*, the striatum likely makes a domain-independent contribution to learning in both cases. Indeed, their analysis demonstrated that what distinguished WM training from perceptual-motor learning was the engagement of the dorsolateral and ventrolateral prefrontal cortex in the former process, although higher striatal and ventrolateral prefrontal activations coupled with lower activation in the dorsolateral prefrontal cortex were better predictors of transfer to other untrained WM tasks. Echoing Dahlin et al. (2008), these results suggest that “the functional roles of the transfer-related regions showing enhanced brain activity suggest that near transfer may not be based on modulation of core WM processes, but on the development of relatively task-specific skills” (Salmi et al., 2018, p. 119).

### Increases vs. Decreases in Activation

When we examined the neural correlates of WM training separately for foci that had exhibited increases vs. decreases in fMRI studies, our results demonstrated that WM training is associated exclusively with decreases in brain activation in clusters within the fronto-parietal system that underlie WM, including bilateral inferior parietal lobule (BA 39/40), middle (BA 9) and superior (BA 6) frontal gyrus, and medial frontal gyrus bordering on the cingulate gyrus (BA 8/32). This observation was somewhat surprising, given that all three previous meta-analyses of WM training had revealed an admixture of activity increases and decreases in the brain (Li et al., 2015; Salmi et al., 2018; Pappa et al., 2020). There could be a few explanations for the divergence of our results with previous meta-analytic studies. First, we opted to focus exclusively on samples of neurologically healthy adults with mean age <65 years, given the well-established finding that older adults display overactivation in functional brain imaging studies, likely as a compensatory mechanism against age-related decline (Reuter-Lorenz and Cappell, 2008; see also Cabeza et al., 2018; Tagliabue and Mazza, 2021). We opted not to focus on the elderly to reduce that possible source of variability in our findings. It is possible that not including those studies may have impacted our findings, although there has been quite a bit of heterogeneity in findings involving the elderly as there have been reports of both increases (Kim et al., 2017; Takeuchi et al., 2020) as well as decreases (Brehmer et al., 2011; Heinzel et al., 2016) in brain activity in relation to WM training. An additional reason might be the choice of training and/or target tasks that formed the focus of our analysis. In terms of the former, it is possible that WM training tasks that target updating might facilitate increases in brain activity in regions that underlie learning of skills and strategies (Pappa et al., 2020). In turn, extending the pre- and post-training measures to tasks that measure other abilities aside from WM (e.g., multitasking and divergent thinking) might engage structures that exhibit increases in brain activity due to the cognitive requirements of those tasks (Salmi et al., 2018). Our findings combined with those of others suggests that even when the focus of the meta-analysis is largely on the same literature, the specific choice of studies can have a noticeable effect on findings, and should be taken into consideration when drawing inferences from the work.

One possible lens for interpreting the reductions observed in brain activation in relation to WM training is in terms of increased expertise. Specifically, it could be argued that repeated practice on the same task, especially in cases where the task was adaptive, likely resulted in greater proficiency in the maintenance and manipulation of information in WM, and that this greater proficiency (i.e., expertise) was reflected in reductions in the BOLD signal in the fronto-parietal WM network. Here we can ask whether expertise is reliably associated with reductions in neural activation across domains. Neumann et al. (2016) conducted an ALE meta-analysis exploring the neural correlates of cognitive expertise in several domains (mental calculation, chess, language, memory, and music without motor involvement), and found that compared to non-experts, experts were more likely to exhibit activation increases rather than decreases. It is important to note that in the studies analyzed by Neumann et al. (2016), persons needed to have had many years of training to qualify as true experts in a domain. It is therefore possible that short-term increases in skill acquisition might lead to reductions in brain activation, whereas true expertise that typically emerges following long-term engagement with domain-specific tasks eventually leads to increases in brain activation (see Klingberg, 2010). In addition to a focus on increases and decreases in brain activation, it is also important to note that in domains such as music, skill learning and expertise are associated not only with increases and decreases in brain activation but also with cortical reorganization, including the formation of new functional connections between brain regions (see Chang, 2014). Although the focus of the present meta-analysis has been on differences in the direction of activations, examining changes in the connectivity of large-scale brain systems and structures in relation to WM training can certainly add to our understanding of its neural bases.

### Impact of Working Memory Training on Span

Although our focus was on the neuroanatomy of WM training, we were also interested in examining whether the studies reported transfer to measures of WM span. We reviewed the results separately for studies of simple vs. complex span, given that they draw on different processes (Unsworth and Engle, 2006). Seven studies from the identified subsample administered measures of simple WM span at pre- and post-test. In the case of three studies, WM training led to statistically significant gains in WM capacity (Olesen et al., 2004; Dahlin et al., 2008; Chang et al., 2017, Experiment 2). A common feature of the training regimens in all three studies was that the task was adaptive, meaning that the level of difficulty was adjusted automatically to maintain maximal cognitive exertion. In contrast, Jolles et al. (2010) and Clark et al. (2017b) who did not use an adaptive version of a WM task found no transfer effect to simple WM span. Finally, Emch et al. (2019) did find a statistically significant Group (Training vs. Control) × Time (pre- vs. post-training) interaction effect, but the interpretation of this effect is complicated by the fact that the performance increase in the experimental group was paired with a performance decrease in the control group. On balance, it seems that when the WM training task is adaptive, then there is a higher likelihood of transfer to simple WM span. In turn, when we switch to complex WM span, there is simply insufficient evidence to infer whether one can observe transfer or not. Specifically, Clark et al. (2017b) found no effect of training on AOSPAN. Furthermore, Flegal et al. (2019) found no effect of training on AOSPAN (Unsworth et al., 2005) or with a change localization (Gold et al., 2006) version of the Change Detection task (Luck and Vogel, 1997), although as noted earlier their focus during training was on WM updating rather than on expanding WM span itself. On balance, it would be prudent to conclude that more research is needed to determine whether WM training can transfer to complex WM span (see also Harrison et al., 2013).

Two additional points deserve attention here. First, as noted by Bryant and Niall (2020), training can impact performance in many ways, such as increasing the power of a cognitive capability, increasing the effect one can derive from an existing level of capacity, and providing external devices to perform cognitive tasks that reduce the need for using cognitive capabilities. In turn, not all of those training outcomes would be equally likely to impact WM capacity *per se*, such that one might observe improvements in WM performance that are not necessarily accompanied by gains in WM span. Second, as noted earlier, there is some evidence to suggest that WM training can lead to near transfer, but there is no such evidence regarding far transfer (Morrison and Chein, 2011; Melby-Lervåg and Hulme, 2013; Redick et al., 2015; Melby-Lervåg et al., 2016; see also Soveri et al., 2017). Although that specific question was not under investigation here, a similar picture emerged across the 32 studies included in our meta-analysis. Pappa et al. (2020) who examined that question formally by conducting a meta-analysis of the behavioral data associated with neuroimaging studies of WM updating found a moderate and statistically significant effect for near transfer (Hedge’s g = 0.63), but a small and statistically non-significant effect for near transfer (Hedge’s g = 0.15). These relatively weak transfer effects likely have a bearing on the neuroanatomy of WM training insofar as one might expect that more robust neural changes would accompany more robust behavioral/performance changes. As the size of this literature grows, it would be important to compare the impact of WM training for studies that report successful vs. unsuccessful near- and far-transfer effects.^5^

### Working Memory Training and Cognitive Resources

Typically, reductions in brain activation in relation to WM training have been attributed to neural efficiency. However, as noted by Poldrack (2015), one could argue for neural efficiency only if the same neural computations were being performed with identical time and intensity, but with different metabolic expenditure. Unfortunately, due to our incomplete understanding of the cellular basis of the BOLD signal (Logothetis, 2008), coupled with the fact that we cannot rule out other factors with certainty (e.g., whether different set of cognitive processes and/or neural computations are being performed), we are not in a position to equate reductions in brain activation in relation to WM training within the fronto-parietal system to neural efficiency (Constantinidis and Klingberg, 2016). Nevertheless, it is prudent to consider the contribution of several candidate processes to this pattern of findings. First, it is possible that the observed pattern is driven by a shift from controlled to automatic processing (Shiffrin and Schneider, 1977). Specifically, it is well known that engagement with an initially novel task can be more effortful, whereas repeated engagement and familiarization with the same task can lead to greater levels of automaticity in task performance. This transition from controlled to automatic processing is captured by dual-process models of cognition that involve an interplay between effortful and automatic processing in the service of task performance (Evans and Over, 1996; Sloman, 1996; Kahneman and Frederick, 2002). In this sense, it is possible that the reduction in brain activation due to WM training could be due to greater automaticity in WM performance because of familiarization (see Chein and Schneider, 2005). Second, decreased brain activity could reflect increased specificity and precision for detecting stimuli—what has been referred to as *narrowing of tuning curves* (Rainer and Miller, 2000). As noted by Constantinidis and Klingberg (2016), a narrower tuning curve could be an indicator that fewer prefrontal or parietal neurons are necessary for coding a stimulus, which will be associated with a lower BOLD response. A third possibility is of course that fewer neurons are engaged for performing the same task post-training—a possibility that has not been tested directly in this domain.

Finally, what do the findings mean for our understanding of WM capacity as a *processing resource* (i.e., an entity that exists in limited supply and is responsible for the enhancing or enabling cognitive processes, Salthouse, 1990)? Historically, scholars who have considered the psychological reality of limited processing resources (or “mental energy”) have typically also assumed that those resources have a physiological correlate (see Craik and Byrd, 1982). In this sense, one would expect that if WM training were to increase WM capacity, then there should be a corresponding change in activity in the neurological system that supports it. Although WM training leads to decreases in activation in the fronto-parietal system, it is not possible to infer that this reflects an increase in WM capacity *per se*. Not only is more research needed to examine how variations in the activity of the fronto-parietal system are related to variations in WM capacity measures, but it is also necessary to consider the broader context within which limited processing resources are measured. As noted by Navon (1984) in his classic criticism of resource models, “resource theory ascribes variability in performance of a task to the amount of some limited *internal input* dedicated to the task” (italics added, p. 217). However, we now know that performance on such tasks and our subjective assessments thereof (e.g., workload) are influenced by a host of contextual, environmental, and motivational factors that likely interact with those internal inputs dedicated to the task. In this sense, examining the neural correlates of constructs hypothesized to be limited by processing resources needs to be informed better by the assumptions that characterize their measurement.

## Conclusion

Our meta-analysis demonstrated that WM training is associated with reduced activation in a set of regions that reside within the fronto-parietal system, including the bilateral inferior parietal lobule (BA 39/40), middle (BA 9) and superior (BA 6) frontal gyrus, and medial frontal gyrus bordering on the cingulate gyrus (BA 8/32) (Figure 2 and Table 3). This pattern of findings suggests that WM training targets neural structures that are involved in the storage, rehearsal, and/or manipulation of mental representations within the core fronto-parietal system that supports WM. Importantly, due to our incomplete understanding of the cellular processes that underlie the BOLD signal, coupled with the fact that we cannot rule out other factors with certainty (e.g., whether different sets of cognitive processes and/or neural computations are being performed), it is not possible to isolate a specific mechanism that can explain the biological basis of the observed reduction in brain activation as a function of WM training. When viewed in the context of extant meta-analytic evidence suggesting that WM training reduces brain activation within the fronto-parietal system (Li et al., 2015; Salmi et al., 2018; Pappa et al., 2020), our results underscore the importance of developing paradigms to examine the biological basis of the observed effect, and thus lead to an improved understanding of what this finding means for resource models of WM.

## Data Availability Statement

The original contributions presented in the study are included in the article, further inquiries can be directed to the corresponding author.

## Author Contributions

OV, VR, SF, and QL contributed to the conception and design of the study. VR, SF, QL, and SS organized the database. OV, VR, SF, QL, and SS performed the statistical analysis. OV wrote the first draft of the manuscript. All authors contributed to the refinement of the design, manuscript revision, read, and approved the submitted version.

## Author Disclaimer

The opinions or assertions contained herein are the private views of the authors and are not to be construed as official or reflecting the views of the U.S. Army or the Department of Defense. Any citations of commercial organizations and trade names in this report do not constitute an official Department of the Army endorsement of approval of the products or services of these organizations.

## Conflict of Interest

The authors declare that the research was conducted in the absence of any commercial or financial relationships that could be construed as a potential conflict of interest.

## Publisher’s Note

All claims expressed in this article are solely those of the authors and do not necessarily represent those of their affiliated organizations, or those of the publisher, the editors and the reviewers. Any product that may be evaluated in this article, or claim that may be made by its manufacturer, is not guaranteed or endorsed by the publisher.

## Footnotes

1 It is important to note that some WM models such as Baddeley’s include “slave” systems for processing modality-specific visual and auditory input, such as the visuospatial sketchpad and the phonological loop, respectively (see Van Erp et al., 2020). We are not making the argument here that such modality-specific systems do not engage the fronto-parietal system.

2
http://brainmap.org

3 Based on a largescale simulation of meta-analysis datasets using empirical parameters derived from the BrainMap database, Eickhoff et al. (2016) demonstrated that cluster-level family-wise error correction represents the most optimal statistical thresholding method, although voxel-wise family-wise error correction also represents an appropriate, but more conservative approach, to statistical thresholding. In turn, both uncorrected inference and correction for multiple comparisons using the false-discovery rate represent inappropriate methods.

4 Note that it was not possible in ALE to directly conduct a contrast analysis involving foci that had exhibited activation decreases vs. foci that had exhibited activation increases because in the latter case no statistically significant results emerged.

5 When using the most optimal thresholding method (i.e., cluster-level family-wise error correction), a minimum of 17 experiments is necessary to perform reliable and robust meta-analyses (Eickhoff et al., 2016).
